# Supplementary material for: Evaluation of Two Simultaneous Metabolomic and Proteomic Extraction Protocols Assessed by Ultra-High-Performance Liquid Chromatography Tandem Mass Spectrometry
Source: Int J Mol Sci. 2023 Jan 10;24(2):1354. doi: 10.3390/ijms24021354 (PMC9865896; doi:10.3390/ijms24021354)
Supplement: Supplementary file 1 [file ijms-24-01354-s001.zip › ijms-2129905-supplementary.pdf]

Supplementary Figure S1

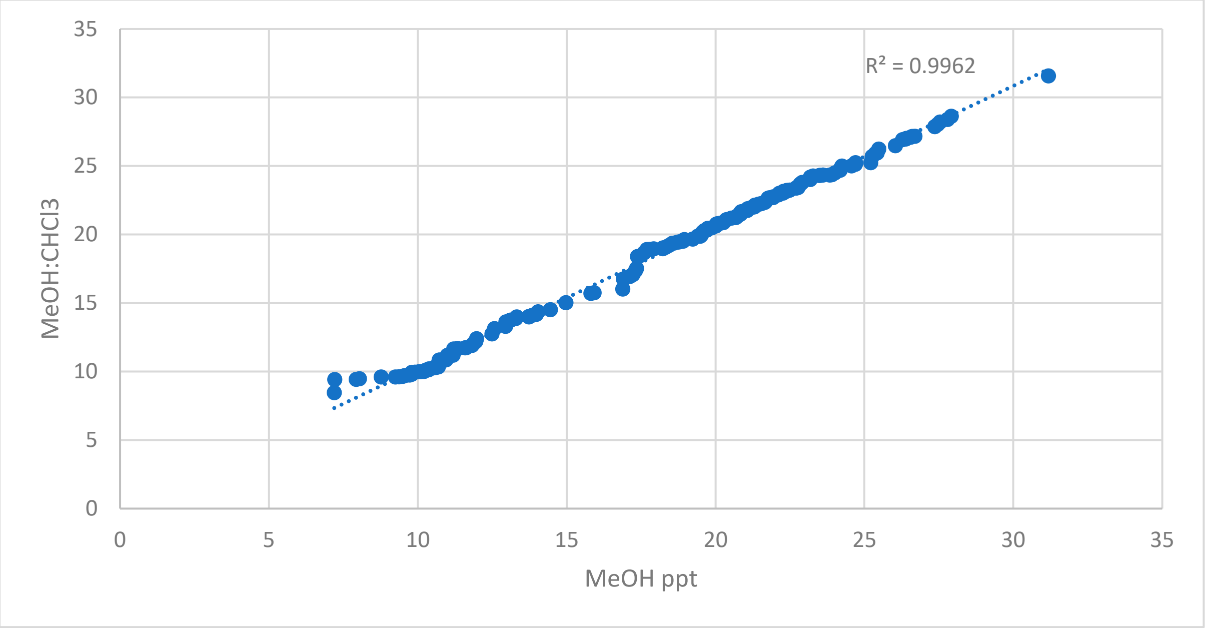

Figure S1. Scatter plot that presents the correlation between the metabolites and proteins for both extraction methods, X-axis indicates log2-transformed average intensities of metabolites and proteins extracted by MeOH-Only Method. Y-axis indicates log2-transformed average intensities of metabolites and proteins extracted by MeOH:$CHCl_3$ Method.
